# Detection of circulating tumour cells in peripheral blood with an automated scanning fluorescence microscope

**DOI:** 10.1038/sj.bjc.6604545

**Published:** 2008-08-05

**Authors:** T G Ntouroupi, S Q Ashraf, S B McGregor, B W Turney, A Seppo, Y Kim, X Wang, M W Kilpatrick, P Tsipouras, T Tafas, W F Bodmer

**Affiliations:** 1 Department of Molecular Oncology, Cancer and Immunogenetics Laboratory, Weatherall Institute of Molecular Medicine, Oxford University, Oxford OX3 9DS, UK; 2 The Centre for Innovation, The Spinal Cord Society of New Zealand, 87 St David Street, PO Box 56, Dunedin 9054, New Zealand; 3 Department of Urology, The Churchill Hospital, Oxford OX3 7LJ, UK; 4 Ikonisys Inc., 5 Science Park, New Haven, CT 06511, USA

**Keywords:** circulating tumour cells, automated fluorescence microscope, colorectal, prostate and ovarian cancer

## Abstract

We have developed an automated, highly sensitive and specific method for identifying and enumerating circulating tumour cells (CTCs) in the blood. Blood samples from 10 prostate, 25 colorectal and 4 ovarian cancer patients were analysed. Eleven healthy donors and seven men with elevated serum prostate-specific antigen (PSA) levels but no evidence of malignancy served as controls. Spiking experiments with cancer cell lines were performed to estimate recovery yield. Isolation was performed either by density gradient centrifugation or by filtration, and the CTCs were labelled with monoclonal antibodies against cytokeratins 7/8 and either AUA1 (against EpCam) or anti-PSA. The slides were analysed with the Ikoniscope® robotic fluorescence microscope imaging system. Spiking experiments showed that less than one epithelial cell per millilitre of blood could be detected, and that fluorescence *in situ* hybridisation (FISH) could identify chromosomal abnormalities in these cells. No positive cells were detected in the 11 healthy control samples. Circulating tumour cells were detected in 23 out of 25 colorectal, 10 out of 10 prostate and 4 out of 4 ovarian cancer patients. Five samples (three colorectal and two ovarian) were analysed by FISH for chromosomes 7 and 8 combined and all had significantly more than four dots per cell. We have demonstrated an Ikoniscope® based relatively simple and rapid procedure for the clear-cut identification of CTCs. The method has considerable promise for screening, early detection of recurrence and evaluation of treatment response for a wide variety of carcinomas.

The detection of circulating tumour cells (CTCs) in peripheral blood was first suggested more than a century ago (Ashworth, 1869), but has only recently become a clinical reality. It is now clear that cells are shed from tumours well before metastasis (Liotta *et al*, 1974), and, therefore, that detection of CTCs could provide a novel approach to screening, detection of recurrence and evaluation of treatment response for many cancers (Cristofanilli and Mendelsohn, 2006; Hayes *et al*, 2006; Cristofanilli *et al*, 2007).

Many methods have been proposed for the detection and enumeration of CTCs including flow cytometry, nucleic acid-based approaches and selective isolation followed by immunofluorescence microscopy (Baran *et al*, 1998; Racila *et al*, 1998; Kowalewska *et al*, 2006; Pinzani *et al*, 2006).

We have developed a novel approach for the identification and characterisation of CTCs based on a combination of antibody fluorescence detection with fluorescence *in situ* hybridisation (FISH), and the use of a fully automated fluorescence microscope (Ntouroupi *et al*, 2007).

## Materials and methods

### Cell lines

The colorectal carcinoma (CRC) cell line C32 (Browning *et al*, 1993) and the prostate carcinoma cell line LNCaP (Horoszewicz *et al*, 1983) were cultured as previously described. Other cell lines were from the Cancer and Immunogenetics laboratory collection (Liu and Bodmer, 2006). Cell counts were determined by a Cellometer® automatic cell counter (Nexcelom Bioscience, Lawrence, MA, USA) and 5–1000 cells were spiked into donor blood to estimate recovery yield. Alternatively, 1–3 cells were micropipetted into blood. All spiking experiments were performed in triplicate.

### Blood samples

Blood samples were obtained from 10 biopsy-proven prostate cancer patients, 25 colorectal cancer and 4 ovarian cancer patients. In addition, blood was collected from seven individuals with elevated serum prostate-specific antigen (PSA) levels but no evidence of malignancy upon biopsy. Blood samples were collected prior to, and within a few weeks of the pre-operative biopsy. The blood samples collected from 11 healthy donors (age ranging from 21 to 71 years old) were used for spiking experiments and as normal controls.

Informed consent was obtained from all patients and donors participating in this study. Investigations were performed after approval by the appropriate research ethical committee.

### Isolation of CTCs by Lymphoprep™

Whole blood was collected in acid citrate dextrose as anticoagulant, and the mononuclear cells, which included epithelial cells, were separated by centrifuging through Lymphoprep (Ficoll-Isopaque, Axis-Shield, Oslo, Norway). The resulting cell suspension (in phosphate buffer saline (PBS), pH 7·4) was deposited on poly-L-lysine-coated, single-well chamber slides (IkoniSlide; Ikonisys Inc., New Haven, CT, USA) at a concentration of approximately 8 × 10^5^ cells per slide. The cells were then fixed with ice-cold methanol for 5 min followed by 2% formaldehyde in PBS for 5 min, washing with PBS (2 × 5 min) and then with PBS-T (0.05% Tween-20 in PBS).

### Isolation of CTCs by filtration with track-etched membranes

Blood was collected into acid citrate dextrose, diluted into 10 volumes of 1 × ammonium chloride-based lysing buffer (BD Pharm Lyse™) containing 0.5% formaldehyde and incubated at room temperature (RT) for 15 min. A Nucleopore (Whatman) track-etched polycarbonate membrane filter (8.0 *μ*m pore size, 25 mm diameter) was placed shiny side up in a reusable syringe filter holder (PALL Life Sciences, Ann Arbor, MI, USA) and a syringe barrel was used as a funnel (Seal, 1964; Song *et al*, 1971). The use of a vacuum manifold (VM20; Sigma, St Louis, MO, USA) allows processing of 20 samples simultaneously. Suction was applied by a vacuum pump briefly at the beginning of the filtration and then the sample was allowed to flow through by gravity. The vacuum produced by the Misrosart® maxi.vac pump (Sartorius, Goettingen, Germany) can be monitored and controlled, thereby allowing the collection of CTCs on the membrane filter under optimal conditions. The diluted blood was filtered through the Nucleopore membrane and washed with 100 ml PBS. The cells retained on the filter were fixed with ice-cold methanol for 5 min followed by 2% formaldehyde in PBS for 5 min. After washing with 100 ml PBS, the filter was removed from the filter holder and processed for antibody staining as described below. This procedure allows fast and efficient isolation of epithelial cells with minimum manipulation, thereby maintaining cellular integrity.

### Antibody staining

All incubations were performed at RT, in humidified chambers, protected from light. The cells were first incubated for 30 min in a blocking solution containing 0.01 g ml^−1^ blocking reagent in PBS-T (TSA™ kit; Molecular Probes Inc.™, Eugene, OR, USA), followed by 30 min incubation with 2 *μ*g ml^−1^ AUA1 in blocking solution. AUA1 is a mouse anti-EpCam monoclonal antibody (Epenetos *et al*, 1982). For some prostate cancer patients, a mouse monoclonal antibody against PSA (ER-PR8; Abcam, Cambridge, MA, USA) was used at a concentration of 10 *μ*g ml^−1^ in blocking solution. Specific binding was detected using HRP-conjugated goat anti-mouse antibody (5 *μ*g ml^−1^ in blocking solution, 30 min) followed by green-fluorescent Alexa Fluor 488 tyramide labelling according to the manufacturer's protocol (TSA; Molecular Probes Inc.). Peroxidase activity was quenched by incubating for 30 min with 2% H_2_O_2_ in PBS-T. After washing with PBS-T, the cells were incubated for 30 min with 7 *μ*g ml^−1^ of the biotinylated mouse monoclonal antibody Cam5.2 against cytokeratins 7/8 (Makin *et al*, 1984). Specific binding was detected using HRP-conjugated streptavidin (5 *μ*g ml^−1^ in blocking solution, 30 min) followed by labelling with far-red fluorescent Alexa Fluor 647 tyramide (which is spectrally similar to Cy5). Specimens were coverslipped with Vectashield mounting medium (Vector, Burlingame, CA, USA) containing DAPI (4′, 6′ diamidino-2-phenylindole), to preserve fluorescence and counterstain the DNA. The use of the Tyramide signal amplification detection method (Molecular Probes Inc.) enables the combination of immunostaining with FISH, without substantial loss of the antibody fluorescence signal intensity. The fluorescent dye-labelled tyramide derivatives are activated by the HRP conjugated to the secondary antibody and are covalently coupled to nucleophilic residues in the vicinity of the HRP–target interaction site. The tyramide signal amplification results in increased sensitivity of detection and stronger signals. In addition, the covalent nature of the binding of fluorescent dyes to targets through tyramide renders the signals more stable and resistant to the conditions the specimens are subjected to during FISH.

### Fluorescence *in situ* hybridisation

Slides with positively immunolabelled cells on them were dehydrated in ethanol series (50, 75 and 100% ethanol, 30 s each), air-dried at 37°C for 15 min and subsequently incubated at 37°C for 3 min in a pre-warmed solution containing 0.001% pepsin and 10 mM HCl. After washing with 50 mM MgCl_2_ in PBS for 5 min, cells were fixed for 10 min at RT with a solution of 2% formaldehyde and 50 mM MgCl_2_ in PBS. After washing in PBS (2 × 5 min, RT) and 2 × SSC (15 min, 37°C), the slides were dehydrated in ethanol series and air-dried. Chromosome enumeration probes (CEP) for chromosomes 7 (aqua), 8 (aqua), 17 (orange) and 18 (aqua) were mixed with hybridisation buffer, and denaturation and hybridisation were performed according to the manufacturer's instructions (Vysis, Downers Grove, IL, USA). Following overnight hybridisation, the slides were washed in pre-warmed 0·4 × SSC buffer with 0·3% NP-40 for 3 min at 72°C followed by 2 min in 2 × SSC with 0·1% NP-40 and 5 min in 2 × SSC. After air-drying for 2 min, the specimens were coverslipped with DAPI-containing Vectashield mounting medium.

### Robotic fluorescence microscopy

Identification and quantification of immunolabelled cells and FISH analysis were performed using the Ikoniscope® imaging system (Kilpatrick *et al*, 2004; Evans *et al*, 2006; Ntouroupi *et al*, 2007). The Ikoniscope robotic, high-throughput, image acquisition and display microscopy system, is developed by Ikonisys for rare cell identification and analysis. It uses epifluorescence optics manufactured by Olympus (Tokyo, Japan). Slides are fed to the instrument through an automated slide/cassette feeder that provides unattended handling of 175 slides. The microscope stage is built for high speed and accuracy of slide movement in each of the *x*, *y* and *z* directions. Image capture is performed through a high-resolution and high-sensitivity monochrome charge-coupled device camera (Hamamatsu Orca ER; Hamamatsu Photonic Systems, Bridgewater, NJ, USA). Carefully controlled exposure setting and automated focusing, combined with three-dimensional image acquisition, are essential for rare cell detection. Cell identification takes place in real time, using image analysis for the detection and quantification of antibody and FISH signals. Preparations are first scanned at low magnification ( × 10) to identify cells carrying both immunolabelled markers. Selected target cells are then revisited at high magnification ( × 100) for verification and enumeration of FISH signals. Results are displayed using the IkoniLAN® viewer software that allows evaluation of low-magnification images from all scanned fields as well as high-magnification images of target cells in all fluorescence channels. All stored information, raw images, processed images and processing results are made available to the reviewers through the IkoniLAN server both in local area computer networks as well as wide area networks using the internet.

## Results

**Figure 1:**
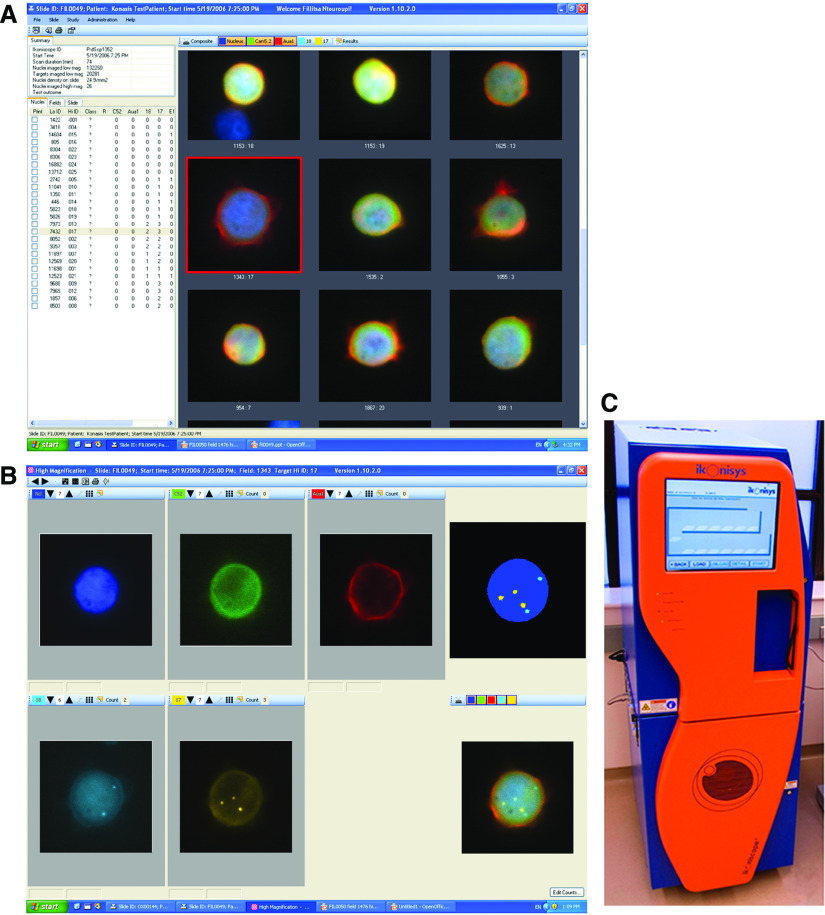
*Screen captures of the Viewer software that displays the data produced by the *Ikoniscope*® scanning system* (**C**). C32 cells were spiked in normal blood, isolated by Lymphoprep^TM^ and immunostained with AUA1 and Cam5.2 followed by FISH with CEP probes for chromosomes 17 and 18. (**A**): High magnification ( × 100) target gallery screen showing composite images of C32 cells for AUA1 (Cy5), Cam5.2 (green) and nuclei (DAPI/blue) staining. (**B**): Screenshot displaying a target C32 cell at high magnification. Pseudo colored images in the DAPI (nucleus), green (Cam5.2), Cy5 (AUA1), orange (CEP 17) and aqua (CEP 18) channels are shown on the left side of the screen shot (clockwise from top left corner). A composite image of all five channels is shown at the bottom right of the screen shot. After automatic identification of FISH signals, a pseudo colored composite image of the nucleus, in which chromosomes 17 appear in yellow and chromosomes 18 appear in aqua, is shown at the top right of the screenshot.

Initial assay development was carried out using Lymphoprep processing of normal blood samples spiked with known numbers of cells from the CRC line C32 and other cell lines. Examples of imaged cells from a spiking experiment are shown in Figure 1. Mononuclear cells were deposited on slides, immunostained with antibodies against EpCam (AUA1) and cytokeratins 7/8 (Cam5.2), and FISH carried out for the enumeration of chromosomes 17 and 18, as described in Materials and Methods. Chromosomes 17 and 18 were chosen because C32 cells exhibit trisomy for 17 and are diploid for 18 and therefore can be used to test the specificity of detection by FISH. Between 90 and 100% of cells deposited on slides were detected down to a dilution of one C32 cell micropipetted into 8 ml of blood and similar results were obtained using the LNCaP prostate carcinoma cells. Figure 1 shows screen captures of the Viewer software that displays the data produced by the Ikoniscope scanning system (Figure 1C) (Kilpatrick *et al*, 2004; Evans *et al*, 2006). Figure 1A shows a high magnification ( × 100) target gallery screen displaying images of identified C32 cells. The displayed images are composites of the Cy5/red (AUA1), green (Cam5.2) and DAPI (blue) channels. Figure 1B displays one of the target C32 cells at high magnification ( × 100). The three dots corresponding to chromosomes 17 appear in yellow and the two dots corresponding to chromosomes 18 appear in aqua, which fits in with the known karyotype of C32 cells.

**Figure 2:**
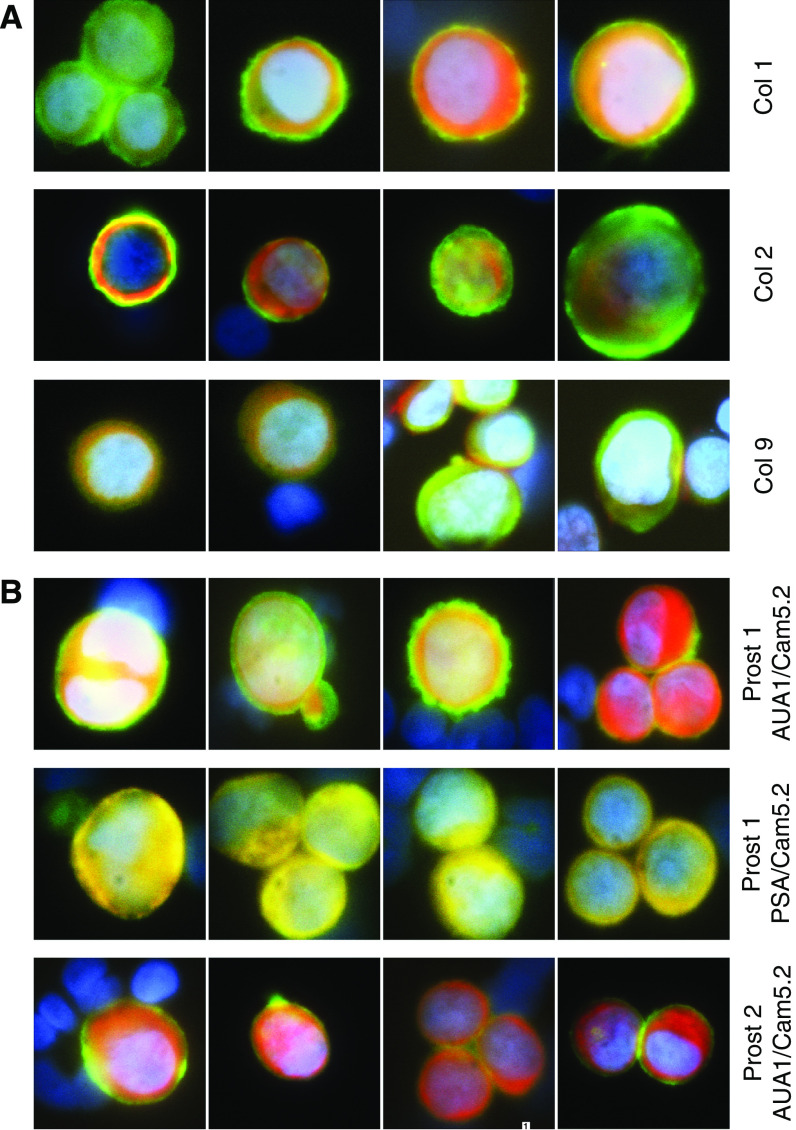
*Circulating tumour cells isolated by Lymphoprep from the peripheral blood of colorectal and prostate cancer patients.* Composite pseudo colored images of cells at high magnification ( × 100) in the DAPI, green and Cy5 channels, are shown. (**A**) CTCs were isolated from colorectal cancer patients Co 1, 2 and 9 and were immunostained with AUA1 (green) and Cam5.2 (Cy5). (**B**) CTCs were isolated from prostate cancer patients 1 and 2 and were immunostained with Cam5.2 (Cy5) and AUA1 or PSA (green), as indicated.

Representative cells identified in blood samples from colorectal and prostate cancer patients, using Lymphoprep isolation and antibody labelling, are shown in Figure 2. These demonstrate the presence of very clearly identifiable doubly labelled epithelial, and so presumptive tumour, cells in both sets of patients.

**Figure 3:**
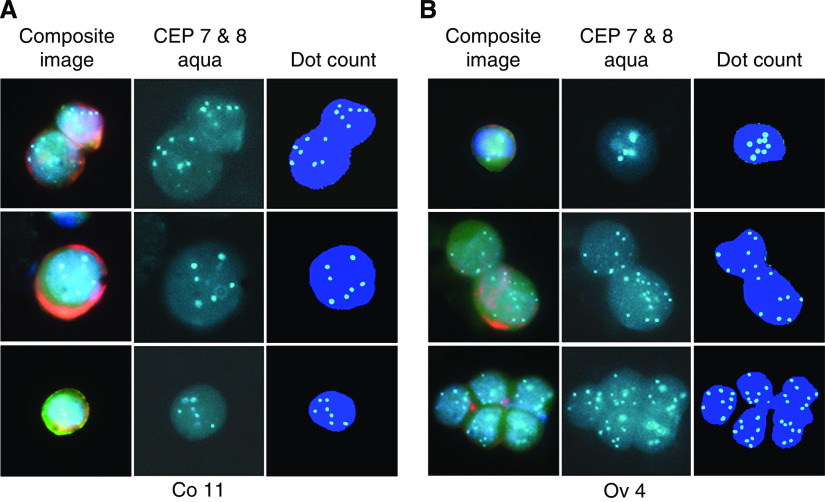
*Circulating tumour cells isolated by filtration from the peripheral blood of colorectal and ovarian cancer patients.* Composite pseudo colored images of cells at high magnification ( × 100) in the DAPI, green, Cy5 and aqua channels are shown on the left of each panel, followed by cell images in the aqua channel in the middle of each panel. After automatic identification of FISH signals (dot count), a pseudo colored composite image of the nucleus, in which chromosomes 7 and 8 appear in aqua, is shown on the right of each panel. (**A**) CTCs were isolated from colorectal cancer patient Co 11 and were immunostained with AUA1 (green) and Cam5.2 (Cy5), followed by FISH with CEP 7/aqua and CEP 8/aqua. A total of 7 aqua dots are present in each nucleus, indicating polysomy for at least one of the chromosomes 7 and 8. (**B**) CTCs were isolated from ovarian cancer patient Ov 4 and were immunostained with AUA1 (green) and Cam5.2 (Cy5), followed by FISH with CEP 7/aqua and CEP 8/aqua. A total of 8 aqua dots are present in each nucleus, indicating polysomy for at least one of the chromosomes 7 and 8.

Spiking reconstruction experiments using the much simpler and more rapid filter procedure, with a range of cell concentrations and 10 different cell lines, gave recoveries of 94–100% (data not shown). This is consistent with measurements of the mean diameters of cells from 20 different cancer cell lines, showing that, on average, more than 90% cells had a mean diameter greater than 8 *μ*m, which is the filter pore diameter (data not shown). Representative cells identified from a colorectal and an ovarian cancer patient as reacting with both the EpCam and cytokeratin 7/8 antibodies, and analysed by FISH for the enumeration of chromosomes 7 and 8 are shown in Figure 3. Each cell is clearly identified by reaction with both antibodies and has a definitely abnormal chromosome count: seven dots for the colorectal and eight dots for the ovarian cancer, compared with the four dots expected. These cells are thus unequivocally identified as CTCs with a readily recognisable morphology. The choice of these particular chromosomes was based on a database search, which showed high incidence of polysomies for chromosomes 7 and 8 in colorectal, prostate and ovarian cancer cell lines and patient cases (http://www.cgap.nci.nih.gov/Chromosomes/Mitelman, http://www.ncbi.nlm.nih.gov/sky/skyweb.cgi).

No cells reacting with both antibodies to EpCam and cytokeratins7/8 have been found in any of the four (two females, two males) Lymphoprep and seven (three females and four males) filter-prepared healthy control samples (Table 1).

A summary of data obtained so far on 25 colorectal, 10 prostate and 4 ovarian cancer patients is shown in Table 1. Presumptive CTCs were not detected in only 2 out of 25 CRC patients, 1 of whom had no nodal involvement. The mean number of CTCs per millilitre was slightly lower (1.6) for the Lymphoprep than for the filter-isolated (2.2) samples.

In all 10 prostate cancer patients analysed, CTCs were detected. Among the seven men who presented with elevated PSA levels but had no evidence of malignancy upon biopsy, four had no detectable CTCs. In the remaining three cases, where the biopsy indicated no malignancy, CTCs were detected (1.4–1.7 cells ml^−1^) strongly suggesting that these patients should be reinvestigated, or at least carefully followed up. It should be noted that in all three of the above-mentioned cases, the identified cells were positive for PSA as well as for AUA1 and Cam5.2. These data, although clearly very preliminary, suggest that the Ikoniscope based CTC detection may be an effective way to reduce the number of false-positives for prostate malignancy based on elevated PSA levels. All four ovarian cancer patients had presumptive CTCs. Table 2 presents the results by patient/control group, indicating the percentage of positive samples as well as the range, mean and median number of CTCs detected per millilitre of blood.

Five patients (three CRCs, two ovarian) were analysed by FISH for chromosomes 7 and 8 combined (see Figure 3) and all had significantly more than four dots per cell.

## Discussion

We have shown that the Ikoniscope based automated fluorescence microscopy can readily detect less than one circulating epithelial cell per millilitre of blood. The use of two different epithelial-specific antibodies, against EpCam and cytokeratin 7/8, makes it most probable that cells, which label with both antibodies, are indeed CTCs. This has been confirmed in those cases where FISH has shown clear-cut chromosomal abnormality, and is made very likely by the fact that no doubly labelled cells were seen in any of 11 normal control samples. The filter isolation procedure is very sensitive and allows the combination of different antibodies and additional downstream analyses (FISH, and so on). The isolation procedure could easily be at least partially automated. Sample manipulation is minimal and the isolation is fast and simple, thereby minimising cell loss and preserving cellular morphology. It also enables all the cells from a 10 ml sample of blood to be deposited on a single slide, which greatly reduces the overall preparation time and cost as well as the microscope scanning time. Currently, the time to scan one slide at both low and high magnification is about 1 h, but in future this will be substantially reduced by anticipated improvements of the Ikoniscope technology. Detection of CTCs by simply counting the cells retained on the filters, as has been proposed (Song *et al*, 1971; Pinzani *et al*, 2006), is clearly not adequate, but when combined with automated immunofluorescence and FISH, identification becomes a highly efficient procedure. The particular advantage of our automated detection system is the ability to image morphologically clearly identifiable cells, which are characterised by both two epithelial-specific antibodies and FISH to detect chromosomal abnormalities, which are a hallmark of most carcinomas.

There are many obvious developments of the procedure we have described. In our study, we used a combination of immunostaining with AUA1/PSA and Cam5.2 followed by FISH for chromosomes 7 and 8 for the identification of CTCs. Future plans including the use of additional antibodies, for example, against p53, MUC-1, the mismatch repair proteins hMLH1 and hMSH2, or cell cycle-specific proteins, may help to define the circulating cells unequivocally as tumour cells even without the need to do FISH. Some antibodies, such as those against cleaved caspase 3 for apoptosis, or against phosphotyrosine residues on signalling proteins, may provide new approaches to non-invasive assessment of response to therapy. Increasing the number of chromosomes that are simultaneously identified by FISH will also help to establish their tumorigenic phenotype. Fluorescence *in situ* hybridisation can also be used to determine HER2, EGFR and other gene amplifications. Eventually, it should be possible to isolate the individual CTCs by, for example, laser microdissection, and then do a more or less comprehensive genetic and gene expression analysis on the isolated cells. The detection strategy may also be used for other sources of cells, for example, from bone marrow, sputum, urine, breast nipple aspirates, lymph nodes or colonic washings.

There are at least three clearly different types of applications for the detection of CTCs. (i) Screening for early detection of cancer. This is probably the most demanding and may often follow on from a cheaper, more high-throughput initial screen, for example, PSA and related tests for prostate cancer, or faecal occult blood for colorectal cancer. However, in the case of ovarian cancer, where so far there has been no satisfactory initial high-throughput screening test, detection of CTCs may well satisfy the needs for a primary screen, perhaps first evaluated in high-risk cases. (ii) Detection of recurrence, where the key is whether the presence of detectable CTCs provides a significant time advantage over other approaches to clinical diagnosis. (iii) Evaluation of response to therapy.

In situations where the aim of CTC detection is to determine whether to proceed with more invasive diagnostic procedures, simply identifying the presence of unexpected epithelial cells in blood may be sufficient. More extensive characterisation of potential cancer cells can then be carried out on biopsy material.

The aim of the present study was to establish proof of principle by analysing relatively small numbers of patient samples from three different cancer types (colorectal, prostate and ovarian). The next step is to study the clinical relevance and utility of our approach to the detection of CTCs, by analysing samples from a large and defined patient cohort.

## Figures and Tables

**Table 1:**
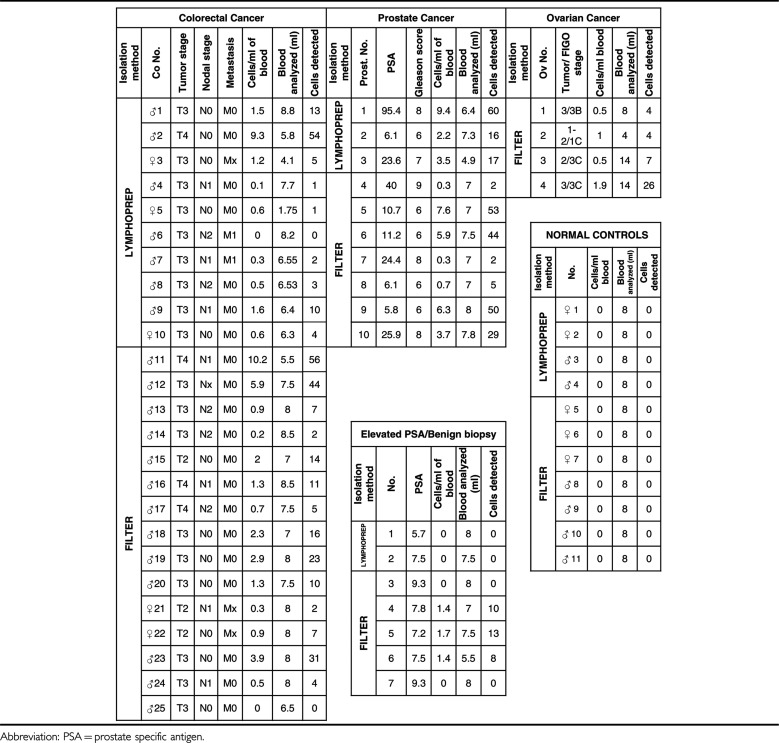
Detection of CTCs in control samples and in colorectal, prostate and ovarian cancer patients

**Table 2:** Summary of results for CTC detection in all sample groups

		**CTC positive**	**Cells per ml blood**		
**Sample group**	***n***	***n* (%)**	**Range**	**Mean**	**Median**
Colorectal	25	23 (92)	0–10.2	1.96	0.9
Prostate	10	10 (100)	0.3–9.4	3.99	3.6
Ovarian	4	4 (100)	0.5–1.9	0.98	0.75
Elevated PSA/NEM	7	3 (42.9)	0–1.7	0.64	0
Healthy controls	11	0 (0)	0	0	0

Abbreviations: PSA=prostate specific antigen; NEM=no evidence of malignancy upon biopsy.
